# Lung cancer with supraclavicular myxoinflammatory fibroblastic sarcoma is easily misdiagnosed as lymph node metastasis: a case report

**DOI:** 10.3389/fonc.2025.1561193

**Published:** 2025-03-28

**Authors:** Qian Yang, Jie Chen, Xiao Feng, Shue Zeng

**Affiliations:** ^1^ Department of Ultrasound, Hubei Cancer Hospital, Tongji Medical College, Huazhong University of Science and Technology, Wuhan, China; ^2^ Department of Oncology, Suizhou Hospital, Hubei University of Medicine, Wuhan, China

**Keywords:** MIFS, lung cancer, lymph node metastasis, misdiagnosis, case report

## Abstract

Myxoinflammatory fibroblastic sarcoma (MIFS) is an infiltrative, locally invasive fibroblastic tumor. A 68-year-old male patient was admitted to the hospital because of a physical examination that revealed a space-occupying lung. Positron emission tomography-CT (PET-CT) showed right upper lung cancer with multiple tiny nodules in both lungs (suspected metastatic foci), and the diagnosis of adenocarcinoma of the right lung was confirmed by aspiration biopsy. In the same period, thyroid nodules were detected by ultrasound and puncture, and papillary thyroid cancer was confirmed by pathology. After multidisciplinary consultation, a systemic treatment plan was drawn up, and changes in the lung nodules were observed. The patient received two cycles of chemotherapy and one cycle of targeted therapy, and the follow-up examination showed shrinkage of the upper lobe of the right lung but stabilization of the intrapulmonary nodule. Still, a mass was visible under the skin on the right neck. Given the abnormal ultrasound of lymph nodes in the V region of the neck and the puncture suggestive of a spindle cell soft tissue tumor, the team of specialists performed radical surgery after a comprehensive evaluation, including resection of the upper lobe of the right lung, systematic lymph node dissection, and enlarged resection of the neck mass. Postoperative pathology finally confirmed that the neck lesion was MIFS. This case suggests that the combination of lung cancer and neck mass should be alerted to the possibility of non-metastatic lesions, especially with supraclavicular lymph node metastasis, which emphasizes the key role of multidisciplinary collaboration and precise pathological diagnosis in the differentiation of complex tumors.

## Introduction

Myxoinflammatory fibroblastic sarcoma (MIFS) is a rare tumor with an annual incidence of less than 1/1,000,000 (1). The first case was reported by Montgomery (2), which is prevalent in adults aged 40–50 years, with a similar male-to-female ratio (3), and is typically manifested as a slow-growing, painless mass in the distal limbs of the hands and feet (3). It presents as a low-grade malignant tumor with a high rate of local recurrence and a low rate of metastasis (4, 5). Notably, MIFS in the supraclavicular region has been rarely reported in the literature. In this paper, we report a complicated case of combined lung and thyroid cancer. The patient was initially diagnosed with suspected metastasis in both lung micronodules and right supraclavicular lymph nodes and was not indicated for surgery for the time being. After chemotherapy and targeted therapy, multidisciplinary evaluation ruled out the possibility of lung metastasis, and the puncture pathology of supraclavicular mass favored the diagnosis of MIFS, and ultimately, radical lung cancer surgery combined with enlarged resection of the neck mass was performed. There were no signs of recurrence at the 18-month postoperative follow-up. This case highlights the importance of accurate differential diagnosis and individualized, sequential treatment in multiple primary tumors. It provides a clinical reference for diagnosing and treating MIFS in rare sites.

## Case history

**Figure 1:**
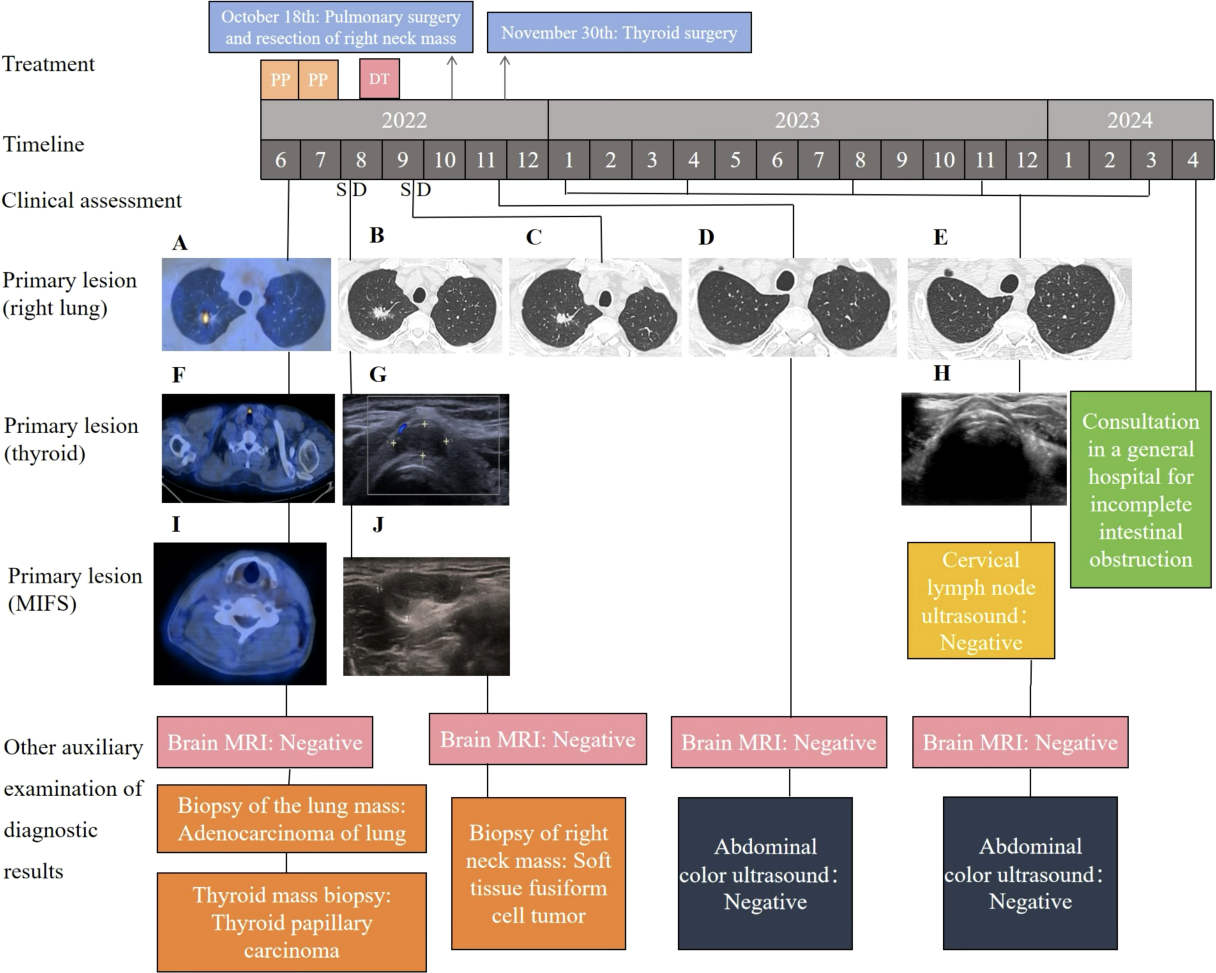
The patient’s treatment history, CT, MRI, color ultrasound, and pathology at various clinical times. **(A)** PET-CT scans showing the primary tumor mass (biopsy site) in the right lung at the time of diagnosis; **(B, C)** chest CT revealing SD following treatments; **(D, E)** chest CT examination after surgery showed no signs of tumor recurrence; **(F)** PET-CT scans showing the primary tumor mass (biopsy site) in the thyroid at the time of diagnosis; **(G)** thyroid color ultrasonography suggests isthmic thyroid nodules; **(H)** postoperative thyroid ultrasound showed no signs of tumor recurrence; **(I)** PET-CT scans showing the primary tumor mass in the right neck subcutaneous at the time of diagnosis; **(J)** the ultrasound results suggested a hypoechoic mass in the V region of the right neck. PP, pemetrexed plus cisplatin; DT, dacomitinib tablets; SD, stable disease. CT, computed tomography; PET-CT, positron emission tomography-CT; MRI, magnetic resonance imaging.

**Figure 2:**
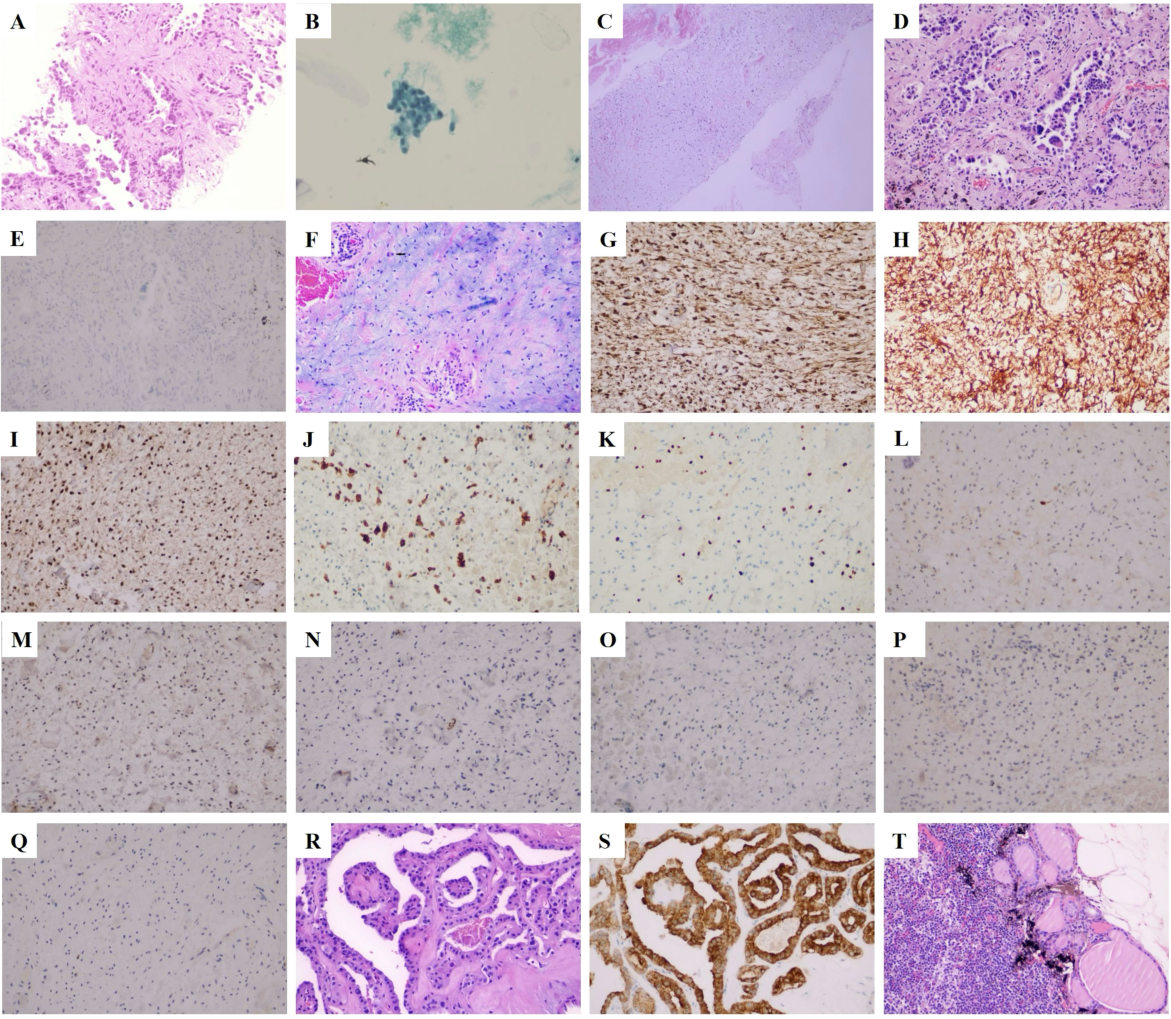
Cell and histopathological features of the tumor. **(A)** A puncture biopsy of the right lung indicated adenocarcinoma (H&E staining, ×200). **(B)** Biopsy of thyroid nodules revealed papillary thyroid carcinoma (Babbitt staining, ×200). **(C)** A puncture biopsy in area V of the right neck indicated a spindle cell soft tissue tumor (H&E staining, ×200). **(D)** Invasive adenocarcinoma of upper lobe of the right lung (H&E staining, ×200). **(E)** ALK negative in the upper lobe of the right lung (IHC staining, ×200; scale bar, 50 µm). **(F)** Histology of the mass in the right neck showed alternating mucoid and fibrous/solid areas with significant inflammatory infiltration and Reed–Sternberg cells (black arrow; H&E staining, 200×). **(G)** Vimentin positive for right neck mass (IHC staining, 200×; scale bar, 50 µm). **(H)** CD34 positive for right neck mass (IHC staining, 200×; scale bar, 50 µm). **(I)** CDK4 positive for right neck mass (IHC staining, 200×; scale bar, 50 µm). **(J)** The CD68 portion of the right cervical mass was positive (IHC staining, 200×; scale bar, 50 µm). **(K)** The ki67 of the right neck mass was 10% (IHC staining, 200×; scale bar, 50 µm). **(L)** S-100 negative for right neck mass (IHC staining, 200×; scale bar, 50 µm). **(M)** MDM2 negative for right neck mass (IHC staining, 200×; scale bar, 50 µm). **(N)** SMA negative for right neck mass (IHC staining, 200×; scale bar, 50 µm). **(O)** BRAF negative for right neck mass (IHC staining, 200×; scale bar, 50 µm). **(P)** SOX-10 negative for right neck mass (IHC staining, 200×; scale bar, 50 µm). **(Q)** PCK negative for right neck mass (IHC staining, 200×; scale bar, 50 µm). **(R)** Papillary thyroid cancer (H&E staining, 200×, 200×). **(S)** Thyroid isthmus carcinoma was BRAF positive (IHC staining, 200×; scale bar, 50 µm). **(T)** Thyroid cancer metastasized to the right central lymph node (H&E staining, 200×, 200×).

A 68-year-old male patient was admitted to the hospital in June 2022 with physical examination findings of correct upper lung occupancy, positron emission tomography-CT (PET-CT) suggestive of right upper lung cancer (**Figure 1A**), multiple tiny nodules in both lungs (maximal diameter <4mm), and thyroid isthmus occupancy (**Figure 1F**). The right upper lung nodule was diagnosed as lung adenocarcinoma (PD-L1 TPS=2%) by computed tomography (CT)-guided puncture (**Figure 2A**), and papillary carcinoma was confirmed by fine-needle aspiration of the thyroid gland (**Figure 2B**).

After a multidisciplinary discussion of lung cancer, from imaging, the patient’s bilateral lung micronodules (diameter less than 4mm) were not a sufficient basis for diagnosing the tumor. Still, bilateral lung metastasis could not be excluded entirely, and it was suggested that systemic chemotherapy should be carried out first, and changes in the bilateral lung nodules should be observed. For papillary thyroid carcinoma, with early staging and good prognosis, close observation is recommended for the time being. From 29 June to 21 July 2022, a Pemetrexed dose of 900 mg plus a cisplatin dose of 130 mg was given for two cycles of chemotherapy. Due to the serious side effects of the second chemotherapy, the chemotherapy was changed to 45 mg targeted therapy with Dacomitinib tablets on 10 August 2022. After 1 week of treatment, the patient developed oral ulcers, and the dose of Dacomitinib tablets was adjusted to 30 mg. On 19 September 2022, the re-examination of the chest CT showed that the upper right lung tumor was smaller than before. There is no noticeable change in double lung nodules. Therefore, the possibility of metastasis of small nodules in both lungs is slight, the nature of the subcutaneous mass in the right neck can be determined, and the possibility of distant metastasis can be ruled out before surgical treatment is considered. The ultrasound results suggested a 2.12 cm ×0.83 cm hypoechoic mass in the V region of the right neck, and the ultrasound-guided puncture pathology suggested a spindle cell soft tissue tumor (**Figure 2C**). Radical right upper lung resection, systematic lymph node dissection, and enlarged resection of the neck mass were performed in October 2022, and the postoperative pathology was clear: (1) there was lung-invasive adenocarcinoma (acinar type) (**Figures 2D, E**), and (2) the right neck mass was consistent with MIFS. Histology showed alternating mucinous and fibrous/solid areas with significant inflammatory infiltrate and atypical epithelioid cells (**Figure 2F**), immunohistochemistry positive for Vimentin (**Figure 2G**), CD34 (**Figure 2H**), CDK4 (**Figure 2I**), partially positive for CD68 (**Figure 2J**), Ki67 index of 10% (**Figure 2K**), and negative for markers such as S-100 (**Figure 2L**), MDM2 (**Figure 2M**), and SMA (**Figure 2N**) (**Figures 2O–Q**). Additional bilateral thyroidectomy lymph node dissection was performed in November 2022, and postoperative staging was pT1bN1aM0 (**Figures 2R–T**).

No evidence of tumor recurrence was seen on chest CT, brain magnetic resonance imaging (MRI), and ultrasonography of thyroid and cervical lymph nodes at 18 months postoperatively. The patient was followed up 1 month postoperatively and every 3 months thereafter. In April 2024, the patient developed incomplete bowel obstruction and was advised to consult a general hospital. The timeline of the patient’s clinical course above is in **Figure 1**.

## Discussion

In our study, we mainly report a case of MIFS with lung cancer combined with thyroid cancer. Ultrasound of the patient’s cervical lymph nodes suggested that a subcutaneous mass in the right neck was not excluded as a supraclavicular lymph node enlargement. The patient then underwent an ultrasound-guided puncture biopsy, and the puncture histopathology results were suggestive of a spindle cell soft tissue tumor. Subsequent pathological findings after complete excision of the patient’s right neck subcutaneous mass suggested that the right neck mass combined with immunohistochemistry (positive for Vimentin, CD34, CDK4, partially positive for CD68, 10% Ki67 index, and negative for markers such as S-100, MDM2, and SMA) further confirmed the presence of MIFS. In recent years, there have been several case-reported studies on MIFS. In 2023, Mohammed I. Alhumaidan et al. reported a case of MIFS in the hand of a 32-year-old male patient who failed to achieve negative margins due to surgery and was treated with postoperative radiotherapy and is undergoing regular follow-up (6). In 2022, Changhong Wei et al. reported a unique case of MIFS in the right parotid gland of a 39-year-old patient who underwent surgical resection and is now in reasonable physical condition. No recurrence or metastasis was found on review (7). However, supraclavicular cases are rarely reported. The rarity of this case lies in (1) the coexistence of MIFS with multiple primary tumors of lung adenocarcinoma and thyroid cancer; (2) the complex histological features of MIFS in the neck (much-fibrotic background with inflammatory infiltration) that need to be differentiated from metastases; and (3) the combination of sequential chemotherapy and targeted therapy with surgery, which highlights the core value of the multi-dimensional integration of pathology–imaging–clinical in the diagnosis and treatment of multiple primary tumors. Diagnosis of MIFS is usually challenging, and the diagnostic process requires a combination of clinical, imaging, and molecular diagnostics. The postoperative histology of our patient showed alternating mucinous and fibrous/solid areas with significant inflammatory infiltration and atypical epithelioid cells, positive immunohistochemistry for Vimentin, CD34, CDK4, partially positive for CD68, a Ki67 index of 10%, and negative markers such as S-100, MDM2, and SMA, which were consistent with the diagnosis of MIFS. MIFS is very easy to be mistaken for other soft tissue tumors, and we summarized the differential diagnosis of MIFS from inflammatory myofibroblastic tumor (IMT) and Giant cell tumor of the tendon sheath (GCTTS) (8–10) (**Table 1**). Various imaging modalities such as X-rays, MRI, and ultrasound are used to detect and follow up the disease. Still, molecular pathology diagnosis of the imaging-guided biopsy is the gold standard (4, 8, 11). MIFS is lobular mainly in shape, varying from gelatinous to firm or fleshy and usually heterogeneous in color and texture (8, 12). Histologically, fibroblast-like cells with spindle shapes, abundant extracellular mucus stroma, inflammatory cells, and chimeric cells are often seen (11, 13). One of the most distinctive histological features of MIFS is the presence of large atypical epithelioid cells, which are usually binucleated or multinucleated, similar to Reed–Stenberg cells or viral cells (14). Immunohistochemically, tumor cells diffusely express Vimentin. Fulfilling both of these points confirms the diagnosis (15). There are fewer cytogenetic studies of this disease, and it has been reported that TGFBR3 and MGEA5 gene rearrangements can be detected by FISH gene testing (16). Another study found a novel TOM1L2-BRAF gene fusion in this disease by targeted sequencing (17).

**Table 1:** Differential diagnosis of MIFS from IMT and GCTTS.

Diagnosis	Key Clinical Features	Imaging Findings	Pathological Features	Immunohistochemistry
Myxoinflammatory Fibroblastic Sarcoma (MIFS)	Adults (30–50 years), distal extremities (hands/feet), slow-growing painless mass	III-defined soft tissue mass; T2-hyperintense (myxoid areas) on MRI	Myxoid stroma, chronic inflammation, Reed–Sternberg-like cells, mild atypia	Vimentin+, CD34±, CD68+; ALK−, S100−, CD15/CD30−
Inflammatory myofibroblastic tumor (IMT)	Children/young adults, visceral/soft tissue	Variable; may show calcifications on CT	Myofibroblastic spindle cells, lymphoplasmacytic infiltrate	ALK+ (50–60%), SMA+, Desmin+; CD34−
Giant cell tumor of the tendon sheath (GCTTS)	Periarticular (knees/hands), slow growing	Lobulated mass with hemosiderin deposits on MRI	Multinucleated giant cells, foam cells, hemosiderin deposits	CD68+, CD163+; SMA−, CD34−

This case occurred in the supraclavicular region, and no cases have been reported at this site nationally or internationally. A preoperative puncture biopsy was performed in this case, but due to the small amount of tissue, classification and diagnosis were difficult, and the final diagnosis still relied on postoperative resection. Mucinous inflammatory fibroblastic sarcoma is locally aggressive. It has a 22%–67% chance of recurrence, but distant metastasis occurs in only 3% of cases (3, 18). The lung is the most common site of distant metastasis in MIFS (18, 19). Unresectable locally advanced or metastatic MIFS (20), atypical histological features (3), and high-grade histology (15) are considered poor prognostic indicators. The treatment of this disease is primarily localized extended resection; in cases with large masses or positive margins, postoperative radiotherapy may be supplemented to reduce recurrence and metastasis rates (19).

## Conclusion

Lung cancer patients with a combined neck mass are highly susceptible to misdiagnosis of supraclavicular lymph node metastasis. Especially in patients with a history of tumor, the possibility of non-metastatic disease should be considered in addition to metastatic disease. Currently, the diagnosis of MIFS relies mainly on clinicopathological diagnosis. In terms of treatment, local enlarged resection is the mainstay; postoperative radiotherapy can be supplemented for cases with large mass sizes or positive margins, thus reducing the chance of recurrence and metastasis. This case provides a valuable paradigm in the diagnosis and treatment of MIFS in rare sites and the integrated management of complex tumors. It also provides an essential reference for subsequent related research and clinical practice.

## Data Availability

The original contributions presented in the study are included in the article/supplementary material. Further inquiries can be directed to the corresponding author.
